# Using the Socioecological Model to Explore Barriers to Health Care Provision in Underserved Communities in the Philippines: Qualitative Study

**DOI:** 10.2196/45669

**Published:** 2023-08-22

**Authors:** Andrew Thomas Reyes, Reimund Serafica, Jennifer Kawi, Miguel Fudolig, Francisco Sy, Erwin William A Leyva, Lorraine S Evangelista

**Affiliations:** 1 School of Nursing University of Nevada Las Vegas Las Vegas, NV United States; 2 School of Public Health University of Nevada Las Vegas Las Vegas, NV United States; 3 College of Nursing University of the Philippines Manila Philippines

**Keywords:** health care delivery, health care access, socioecological model, Philippines, community, barriers, health care, Asian and Pacific Islander, focus group, Tagalog, thematic analysis, socioecological framework

## Abstract

**Background:**

The Philippines’ primary care is delivered via local health centers called barangay health centers (BHCs). Barangays are the most local government units in the Philippines. Designed to promote and prevent disease via basic health care, these BHCs are staffed mainly by barangay health workers (BHWs). However, there has been limited research on the social and environmental factors affecting underserved communities’ access to health care in underserved areas of the Philippines. Given the importance of BHCs in disease prevention and health promotion, it is necessary to identify obstacles to providing their services and initiatives.

**Objective:**

This study aimed to explore multilevel barriers to accessing and providing basic health care in BHCs.

**Methods:**

We used a qualitative approach and the socioecological model as a framework to investigate the multilevel barriers affecting basic health care provision. A total of 18 BHWs from 6 BHCs nationwide participated in focus group interviews. Traditional thematic content analysis was used to analyze the focus group data. After that, we conducted individual semistructured interviews with 4 public health nurses who supervised the BHWs to confirm findings from focus groups as a data source triangulation. The final stage of thematic analysis was conducted using the socioecological model as the framework.

**Results:**

Findings revealed various barriers at the individual (lack of staff motivation and misperceptions of health care needs), interpersonal (lack of training, unprofessional behaviors, and lack of communication), institutional (lack of human resources for health, lack of accountability of staff, unrealistic expectations, and lack of physical space or supplies), community (lack of community support, lack of availability of appropriate resources, and belief in traditional healers), and policy (lack of uniformity in policies and resources and lack of a functional infrastructure) levels.

**Conclusions:**

Examining individual-, interpersonal-, institutional-, community-, and policy-level determinants that affect BHCs can inform community-based health promotion interventions for the country’s underserved communities. Given the multidimensional barriers identified, a comprehensive program must be developed and implemented in collaboration with health care providers, community leaders, local and regional health care department representatives, and policy makers.

## Introduction

In the Philippines, women, older adults, low-income households, and people with disabilities face barriers to basic health care, particularly in underserved areas [1]. The Local Government Code of 1991 (Republic Act no. 7160) decentralized public health care, assets, liabilities, equipment, workers, and records to local governments in the country [2]. By decentralizing care to local government units (called *barangays*), the country aims to improve health care services by bringing them closer to consumers [3]. Decentralization allowed local governments to form organizations, improve and standardize services, and offer a platform for best practices and new laws [4]. The barangay’s decentralized local government budget specifies the number of barangay health workers (BHWs), responsibilities, and salaries or allowances [3].

As a result of decentralization, health centers in barangays (the smallest administrative division in the Philippines) were tasked with providing basic health care (eg, vaccines, health and nutrition education, family planning services, treatment for minor illnesses and injuries) to the individuals residing in the communities they served [5]. These barangay health centers (BHCs) are the cornerstone of the country’s public health system and were designed to promote health and prevent disease by providing basic health care [3]. Unfortunately, barely half of Filipinos can access a BHC within 30 minutes of travel time [6]. Also, previous research has demonstrated that decentralization does not always lead to greater equality, efficiency, and effectiveness in health care delivery. Instead, it can worsen inequality, erode local commitment to critical health issues, and reduce the efficiency and efficacy of health care delivery to underserved populations [2]. Decentralization has also decreased health care quality in some of the country’s poorest local government units [6].

The Philippines’ BHCs and their corresponding BHWs have been considered longer in operation as local government–trained health care providers than in other countries [7]. Therefore, exploring factors that facilitate and hinder the health care service delivery of BHWs may offer interesting insights into improving the health care services provided by local government–trained health care providers in countries that provide decentralized health care services. To enhance health outcomes on all fronts, BHWs are employed at the barangay level as a bridge between health care institutions and local communities to increase access to care [8]. Because most BHWs come from the communities they serve, they are well versed in the issues that the community members face and can tailor their care accordingly. In addition to taking vital signs, BHWs can advise on maintaining a healthy lifestyle, administering basic treatments, and making referrals when necessary [9]. However, there is a lack of knowledge of the motivational factors that make these primarily unpaid volunteers work and the challenges they face in providing access to basic health care to these vulnerable populations [10]. Recognizing these problems is crucial to the country’s primary health care success and sustainability [11].

To address this gap in the literature, we used the socioecological model as a conceptual and organizing framework to investigate barriers to basic health care from the perspectives of BHWs and public health nurses, as direct supervisors of BHWs, in several underserved communities in the Philippines. The socioecological model is well established and can investigate how social and environmental factors across ecological levels (ie, individual, interpersonal, institutional, community, and policy) influence basic health care provision or lack thereof in underserved communities [12]. This model assists in identifying context-specific factors, which are typically overlooked in research, that either reduce or promote access to basic health care.

This qualitative study is significant because many countries, particularly low- and middle-income countries, use decentralized health care services, and the grassroots level of health care accessed by the communities of these countries is those programs provided by community health workers (CHWs) [13], such as the Filipino BHWs in this study. An explorative study investigating factors that facilitate and hinder health care provision by CHWs at all levels (ie, individual, interpersonal, institutional, community, and policy) is imperative. Therefore, we conducted this qualitative study to explore factors affecting the health care provision of BHWs in the Philippines using a socioecological framework. The study’s findings will provide nuanced evidence on the commonly identified challenges of community health care programs in low- and middle-income countries, such as inadequate government funding, lack of supervision and training of community health care providers, insufficient focus on health promotion and prevention, and fragmented programming [14-16]. The study’s findings can also provide the basis for helping local, national, and international stakeholders maximize their support for various community health programs, particularly in countries that use decentralized health care services. The comprehensive approach (ie, socioecological framework) and the qualitative focus of the study will provide findings that can further clarify ambiguous and fragmented challenges that interface between broader health systems and point-of-care services; hence, the results of the study can provide a multilevel basis for overcoming challenges with decentralized health care services in order for community health programs to reach their full potential. Therefore, the purpose of the study is to explore the multilevel barriers to accessing and providing basic health care in community health centers in the Philippines called BHCs.

## Methods

### Study Design

The study team employed a qualitative descriptive approach using focus group sessions and individual interviews. We followed the COREQ (Consolidated Criteria for Reporting Qualitative Research) guidelines [17]. We used 2 sets of semistructured interview guides—one for focus groups and one for individual interviews—to explore our study topic; the conceptual underpinnings of both sets were derived from a literature review. Textbox 1 outlines sample questions included in the semistructured interview guide. The interview guides were provided in Tagalog (the local language) and English. The focus group discussions focused on assessing the experiences and perspectives of BHWs in delivering basic health care at their BHCs and the obstacles they faced in providing health care and using community resources for community members. The focus groups also explored how different levels of government-provided health care to the population. The individual interviews had the same goals but focused on the public health nurses’ perspectives of the BHWs’ attitudes and work conditions and the community’s response to the BHWs’ roles in the BHCs they served.

**The semistructured interview guide.**

**Sample questions for focus groups and individual interviews**

- In your opinion, what was the situation of basic health care in your community, or how accessible was basic health care in your community? Please explain.
- In your observation or experiences, how were you or people in your community using basic health care before the pandemic?
- Please share your experiences or efforts in delivering basic health care in your community. (Only ask health workers, female community health volunteers, and local elected authorities.)
- In your observation or experiences, how are the different levels of governments responding to providing health care to community people?
- Please feel free to share if you have any suggestions or anything you would like to say or think you missed during our conversation.

### Study Setting and Participants

A total of 6 focus groups were interviewed. Each focus group comprised 3 BHWs from the Philippines’ 6 regions, including the National Capital, Cordilleras, Ilocos Region, Central Luzon, Western Visayas, and Central Visayas (N=18). The BHCs of these regions of the Philippines served between 20,000 and 25,000 people [6]. The majority of BHWs (15/18, 83%) were older than 40 years (mean 50.8, SD 9.6 years), and 78% (14/18) had spent more than 6 years in their current occupation (mean 17.1, SD 10.3 years) and workplace (mean 12.9, SD 9.6 years). Additionally, 4 public health nurses (all female, average age 23.6, SD 5.6 years) assigned to supervise BHWs from 3 to 5 BHCs participated in the individual interviews to confirm preliminary findings from our focus group data analysis.

### Sampling and Data Collection

Purposive sampling was used to interview BHWs through focus groups. After all 6 focus group interviews were completed, an initial thematic analysis was conducted to arrive at emerging categories and preliminary themes. After data source triangulation [18,19], these preliminary themes were forwarded to the public health nurses supervising the BHWs through in-person individual interviews. To protect the identity of the BHWs, personal identifying information was not disclosed during the individual interviews. Additionally, the individual interviews did not discuss information from the preliminary themes that could directly refer to the identity of the BHWs. For example, information was more expressed in general terms (eg, information related to the punctuality of a particular BHW was shared in general terms applicable to a larger group of BHWs, such as “Some BHWs in some clinics were often late in coming to work” as opposed to referring to a particular BHW).

A female master’s-prepared nurse who was not part of the study team but was trained and skilled in qualitative research conducted all focus group discussions and individual interviews. The interviewer introduced herself and gave an overview of the research before each interview or group discussion. All individuals were allowed to ask questions and provided consent.

Interviews were in Tagalog or English based on the participants’ request. All interviews were held at the BHCs. All individual interviews and focus groups, which lasted an average of 45 to 60 minutes, were recorded, transcribed, and translated into English. All data were deidentified before transcription and analyses. Two days after transcribing, the interviewer and research team convened to review the transcripts; they did not find any ambiguous questions or topics from the transcripts requiring follow-up interviews.

### Data Analysis

We used a deductive thematic analysis to identify the service gaps, challenges, and constraints to providing health care in the barangays. The gathered data were structured and analyzed using a socioecological framework [12] frequently used as a foundational framework for research in health promotion behavior interventions.

ATLAS.ti (ATLAS.ti Scientific Software Development GmbH) [20] was used to analyze transcripts for themes and patterns. Two bilingual researchers (EWAL and LSE) trained in qualitative research compared transcriptions with original recordings to verify accuracy. Subsequently, these 2 researchers coded the first few transcripts and met with a senior researcher to discuss discrepancies and new topics. After comparing and contrasting the coding, we obtained consensus on the emerging codes and categories and implemented these as a template for coding on the remaining transcripts.

The female master’s-prepared nurse who originally conducted the interviews reviewed the emerging code and categories with the 2 bilingual researchers to organize the themes for the individual follow-up interviews with the nurses (data source triangulation). Statements that could have direct reference to a BHW or could reveal the personal identifying information of the BHW were highlighted to omit them from the discussions in the individual interviews. After all individual interviews were conducted, the final stage of thematic analysis was conducted to include insights from the individual interviews. In the final analysis stage, all identified themes were grouped and categorized into a socioecological model level with no emerging themes outside the socioecological framework.

### Ethics Approval

The University of California Irvine Institutional Review Board and the University of the Philippines Ethics Review Board (IRB approval number: UPMRED #2016-496-01) approved this study. Before conducting any interviews, each participant was informed of the purpose of the study, and we ensured that all participants provided written informed consent.

## Results

### Levels of the Socioecological Model

**Figure 1:**
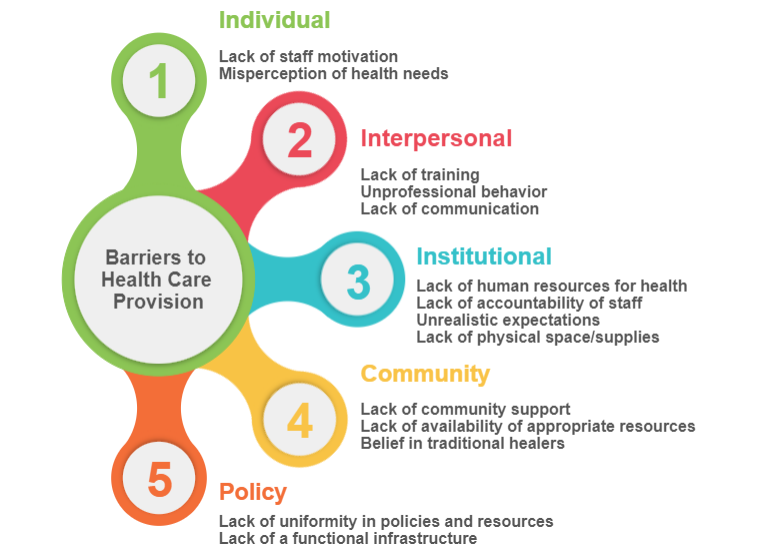
Barriers to health care provision in barangay health centers.

This study aimed to examine barriers to basic health care from the viewpoints of BHWs and public health nurses in several underserved communities in the Philippines. Using the socioecological model framework, we organized the concepts we found into five broad categories: (1) individual, (2) interpersonal, (3) institutional, (4) community, and (5) policy (Figure 1). The socioecological model is a system model based on the notion that social contexts determine the actions and reactions of individuals and that multiple factors influence and are influenced by the behavior of individuals [12]. It highlights the importance of addressing the interaction between individuals and their sociocultural environment at all system levels and the interdependence of influencing factors within and across all health problems and behaviors. Themes are presented according to the 5 levels of the socioecological model and augmented with illustrative quotes (see Table 1).

**Table 1:** Themes with representative quotes.

Levels and themes		Sample quotes
**Individual-level barriers**		
	Lack of staff motivation	“Sometimes, we don’t want to go to the center because there aren't any people to see or because we can’t do anything about their health problems.” (Christina, BHW^a^)
	Misperceptions of health needs	“People in our community don’t go to the center because they don’t think it’s necessary to be seen for their high blood pressure. Instead, they go to the city or provincial hospital when they are really sick.” (Lolita, BHW)
**Interpersonal-level barriers**		
	Lack of training	“Because not everyone got the same training to become a BHW, some of us are less skilled than others. Then there is experience. For example, some of us have worked with local midwives, while others have not.” (Melinda, BHW)
	Unprofessional behaviors	“One of the BHWs working at our center was rude to the patients and their families. Because of this, the center had to close because the people it served went to a nearby BHC for basic care.” (Josephine, BHW)
	Lack of communication	“We never see the nurses, so if we aren’t sure what to do, we have to send them a text message, which they may not answer for several days. So, we send patients home without taking care of them.” (Luninging, BHW)
**Institutional-level barriers**		
	Lack of human resources for health	“There are just days when it is busy, and I can’t take care of all the people coming in. We just need more manpower.” (Magnolia, BHW)
	Lack of accountability of staff	“No one is held responsible for being at work, so no one sticks to the schedule, which slows things down. At the centers, we sometimes work alone, which is frustrating because we can’t get everything done.” (Gwendolyn, BHW)
	Unrealistic expectations	“What we do as BHWs changes from day to day. If community leaders need us, sometimes for personal reasons, we have to leave the center. The nurses who watch over us don’t know what we can do, and some let us do what we feel comfortable with. However, some nurses will ask us to do things we shouldn't, clean wounds.” (Delia, BHW)
	Lack of physical space or supplies	“The space in our BHC is so small that only the BHW, the patient, and one family member can be there at once. Because of this, more people who need healthcare must wait outside, often for long periods. We also don’t have electric fans, so it gets very hot. Our mayor promised to build a bigger and better facility to replace the one we have, but that has been a plan for several years.” (Carmelita, BHW)
**Community-level barriers**		
	Lack of community support	“With the help of our community leaders, we have done projects to reach out to the community. But this is not consistent, and we often feel unsupported.” (Marilou, BHW)
	Lack of availability of appropriate resources	“Our wealthier members will go to hospitals in the city, while those with less money will go to an herbalist or a quack doctor. Most say that the centers don’t have enough resources or supplies for their needs.” (Tomasa, BHW)
	Belief in traditional healers	“Our herbalists and quack doctors have everything they need to treat common illnesses and can give them to sick people for free. Therefore, they are more trusted than we (BHWs) are.” (Amelia, BHW)
**Policy-level barriers**		
	Lack of uniformity in policies and resources	“Depending on how much money the local government spends on healthcare, some BHCs may have more supplies and resources than others. Also, they have more space and supplies. But we can’t be like them if we do not have funds.” (Jocelyn, BHW)
	Lack of a functional infrastructure	“Donors sometimes make donations, but they are very infrequent and local leaders will make promises but not carry them out.” (Marilyn, BHW)

^a^BHW: barangay health worker.

### Individual-Level Factors

Participants shared several individual-level factors that they deemed were barriers to providing basic health care at the BHCs. These individual characteristics were BHW’s self-motivation and community members’ misperceptions of their health care needs. In addition, individual-level characteristics reported by participants were influenced by determinants at all other socioecological model levels, particularly those linked to interpersonal and institutional factors.

### Interpersonal-Level Factors

Participants frequently brought up interpersonal concerns such as a lack of training, unprofessional behavior among peers, and poor communication. BHWs, for instance, have reported feeling unprepared for their roles because of a lack of formal training. The nurses confirmed this BHW’s concern that they could not support the BHWs as much as possible because of their scope of responsibilities of managing more than one BHC. During the confirmatory interviews, nurses also expressed their need to provide closer supervision of the BHWs to help the BHWs develop more confidence in performing basic health care services. Other BHWs reported the opposite, citing informal training with more seasoned peers (BHWs) in their BHCs as the source of a better understanding of their roles and a renewed sense of motivation to perform at their best within the limits of their job. However, BHWs voiced concerns that not all nurses (considered as their supervisors) could be contacted for advice when necessary. As a result, the nurses’ lack of access to supervise and mentor the BHWs contributed to the BHWs’ ongoing frustration and decreased job satisfaction. Lastly, the BHWs rarely had someone check in on their progress because the nurses were often unavailable to supervise them and oversee their performance at the clinics. During the confirmatory individual interviews with the nurses, it was evident that there were differences in perceived priorities between the nurses and the BHWs. For example, BHWs prioritized improving their ability to provide basic health care services. At the same time, the nurses wanted to expand the BHWs’ scope of responsibilities so they could function even without the presence of the nurses in the clinics. Nurses often found resistance from BHWs in their attempts to expand BHW’s scope of practice; this perceived resistance often resulted in more distant communication between the BHWs and the nurses. Therefore, this poor (often nonexistent) communication was detrimental to their functions as primary health care providers.

### Institutional-Level Factors

Participants said that a BHC’s ability to provide basic health care primarily depends on the availability of health workers and the quality of care delivered by staff. Despite this, there was a lack of accountability among BHWs such as only a handful of dedicated BHWs working at the BHCs, with some employees regularly missing work and others complaining about how much they had to do and how little help they got. In addition, basic health care was difficult to deliver for various reasons, such as conflicting and unrealistic expectations from community leaders and members, supervisors, and local government entities. For example, BHCs were promised by community leaders (eg, city mayors and barangay leaders) more funding, but BHWs continued to wait for these plans to be realized; therefore, basic medical supplies were limited, resulting in challenges with delivering basic health care services. In another example, nurses and BHWs were expected to deliver more community outreach programs by local government entities; however, they expressed frustration with the incongruence between the lack of public funding and the increasing expectation to deliver more outreach programs. BHWs voiced that they largely depended on volunteers from private organizations for their outreach programs, and they also articulated that the support they received from these private volunteers was inconsistent and infrequent.

During the group sessions, accessibility to the BHCs (ie, the physical distance between the center and the people it serves) and transportation costs determined whether community members would come for basic health care. It was also emphasized that the infrastructure and resources varied from center to center. Patients frequently had to wait outside the center in the heat and occasionally rainy weather due to a lack of physical space within the BHCs. Individuals were less inclined to seek preventative treatment at the BHCs because of the absence of functioning fans and air conditioning. When the patients obtained the help they needed, there was a limited selection of services and items they could avail themselves of during their visit to the BHC. Free access to essential medical items such as medication, contraception, and water was only offered at a select number of BHCs.

### Community-Level Factors

At the community level, thematic clusters emerged with environmental elements such as the lack of community support, usually demonstrated by informal networks like community leaders influencing basic health care delivery. For example, some BHCs benefited from donations from wealthier community members, but this was uncommon. Another environmental consideration was the availability of community resources. Furthermore, despite efforts to encourage community members to seek basic treatment at BHCs, affluent members would go to city or provincial hospitals related to the lack of appropriate health resources available at the BHC level. On the other hand, the poorer members sought treatment from traditional healers such as an herbalist or quack doctors.

### Policy-Level Factors

Policy topics that emerged were a lack of uniformity in policies governing the provision of essential health care by individual local government bodies. Different towns have varied requirements for providing citizens with basic medical care. Another theme evident among the participants’ interviews was the lack of functional health infrastructure. The health care resources made accessible by each jurisdiction influence the accessibility of basic health care services (such as personnel, supplies or equipment, and medications). Consequently, there was an inadequate understanding of the benefits and expected health care coverage offered by the public and private health care sectors.

## Discussion

### Principal Findings

The provision of primary health care in low- and middle-income countries has been the subject of prior research that used the socioecological model [21-24]. However, no study has examined the perspectives of BHWs and public health nurses who work in BHCs to provide basic health care in underserved regions in the Philippines using the socioecological model as a framework [12]. Our research indicates that basic health care provision in BHCs across the Philippines is influenced by factors at all levels of the socioecological model. The premise of the socioecological model is that health policy decisions and practices affect not only individuals but also the social networks in which they participate and the institutions and communities in which they reside [25].

There are several similarities between this study’s findings and other studies on primary health care services provided by CHWs in low- and middle-income countries. Findings from our study that are consistent with previous research include the influence of the lack of physical space and adequate ventilation on the quality of health care services provided by the CHWs [26], the impact of the quality of the partnership between the local health system and the CHWs on the community perceptions of the quality of services provided by the health centers [27], the relationship between the community’s pervasive traditional beliefs of non-Western medical care and the lack of trust of CHWs [9,28], lack of consistent funding from local health systems [29,30], variability of basic and supplementary training of CHWs among health centers [8], and inconsistent and infrequent funding from private and nongovernmental organizations [8].

However, the area in which our findings are distinct from previous studies on primary health care services provided by CHWs in low- and middle-income countries is the interactional processes between the BHWs and the public health nurses who oversee the work of the BHWs. Although previous studies highlighted the importance of a collaborative working relationship between CHWs and higher-level health workers [15,31-33], these studies did not specifically explore the supervisory role of public health nurses in the work of the CHWs. Our findings specifically demonstrate that the collaboration between the public health nurses and the BHWs is crucial in providing health care services to the BHWs. Because the scope of responsibilities of BHWs is mostly based on nursing functions, supervision, training, and mentoring of BHWs by public health nurses are imperative. Nursing perspectives in the partnership between public health nurses and BHWs are incumbently necessary. The lack of confidence and competence of BHWs in performing their assigned nursing tasks in the clinics was attributed to the lack of formal training, supervision, and mentoring by their nursing supervisors (ie, the public health nurses).

Additionally, our study’s findings show that community members were not maximizing the use of the services offered by the clinics because the services provided by the BHWs were limited. The limited scope of practice of BHWs was mainly related to the lack of availability and limited accessibility of public health nurses to train, supervise, and monitor the progress of their performance. The BHWs required the supervision of the public health nurses to carry out their nursing tasks to expand their scope of responsibilities and be able to perform nursing functions more independently, especially when the nurses are not able to promptly respond to the needs of the clinics (eg, the public health nurses were responsible for overseeing several clinics or health centers). Our findings provide the basis for developing nursing-specific standards and policies in monitoring the progress of BHWs’ performance and expanding their scope of responsibilities to perform more independent functions and respond to rapidly changing and complex clinical situations. Our findings also demonstrate the need to integrate nursing perspectives in training CHWs to deliver point-of-care health care programs to the communities. For example, the type of services and coordination provided by the BHWs are mainly based on nursing; therefore, public health nurses are the most appropriate supervisors, mentors, and coaches for the BHWs. More importantly, the limited resources provided to the BHCs and the increasing scope of responsibilities of public health nurses in managing multiple health centers require BHWs to expand their scope of practice; the public health nurses are the main drivers in the professional development of BHWs to provide timely and safe health care services to the populations they serve.

Our research reveals that poor working conditions and limited resources significantly hinder providing high-quality basic health care in underserved areas. These findings are consistent with earlier research examining health care delivery in underserved areas in the country [1,5,9,11,25]. Given the magnitude of the problems afflicting the health care system in the Philippines, an interdisciplinary and cross-sectoral approach is required to improve the quality of health care provided in BHCs by enhancing their staffing, clinical resources, and access to life-saving drugs [33,34]. The vital role of BHWs within the larger health system should be reflected in resource allocation as the Philippines and other contexts make strides toward universal health coverage.

The absence of supervision and proper training for BHWs prevented them from playing their full role in providing primary care to their communities. BHWs are employed and trained by local government units to support various health programs. Unfortunately, not all BHWs get the training they need. Some BHWs claim that they were not provided with a formal training program and were educated by experienced BHWs on how to perform their duties; if the training was provided at all, its scope, depth, and duration varied across different local communities. Given the disparity in training, BHWs may have difficulty understanding and consistently carrying out their tasks. Moreover, lack of adequate training may lead to the communities’ lack of trust and hesitancy to seek care from BHWs, as revealed in our findings. Our results are consistent with other studies performed in the Philippines [1,9] and elsewhere [35] on BHW initiatives in low- and middle-income countries. For this reason, the Philippine Department of Health must attempt to standardize the training of BHWs throughout the country. The effectiveness of BHC programs and safe, quality health care in the communities depend on BHWs receiving high-quality initial and continuous training.

In addition to standardizing the initial and ongoing training of BHWs, another widely discussed option in the country is to accredit BHW programs that can significantly improve the usefulness of BHC programs, both for their communities and BHWs. The Philippines’ BHW Act (1995) demanded BHW accreditation as a viable tool to increase BHWs’ morale, job security, career chances, legitimacy, and social standing [8]. Accreditation for BHW programs has been shown to protect them from acquiescing to the agenda of local political leaders, ensuring their continued existence [8]. Furthermore, accreditation can increase reliability in executing BHW programs in various settings by improving oversight and standardizing BHW performance [8]. However, previous initiatives to support the accreditation of BHW programs were unsuccessful at the local level in the Philippines.

More research is needed to explore the governance challenges and opportunities of BHW programs in various decentralized health systems to realize the full role BHW programs can play in achieving universal health care. This includes the ability of programs to extend the reach of formal health care providers, enhance access and equity of health care, and improve individual- and community-level health outcomes. In the decentralized Philippines, BHWs could be “local” health experts in multistakeholder talks on planning, financing, implementing, managing, and monitoring community health care. Our research indicates that BHC programs and BHWs can be more effectively leveraged in efforts to achieve universal health care by increasing the capacity of local governments to provide sufficient resources to BHC programs and BHWs [8]. Providing BHWs a voice in policy decisions that pertain to their work might increase transparency and accountability by involving more government agencies (ie, local communities and regional or national health authorities) [36].

Our study has some limitations. Using a convenience sample, for example, increases the possibility of selection bias. In addition, the findings represent the viewpoints of numerous BHWs and nurses working in underserved areas in the Philippines. However, they were mainly from Luzon and Visayas (the northern and mid-regions of the country), making generalization difficult. Moreover, our participants were female, highlighting a crucial gendered aspect of BHWs in the Philippines. In societies where care is feminized, it is crucial to recognize and address gender inequalities to ensure that the work and time of BHWs are adequately appreciated [8]. Nevertheless, the study has numerous merits, such as the iterative process of developing a framework of barriers that hamper the delivery of basic health care to BHCs in the Philippines. Furthermore, this is the first known and documented study to use the socioecological model to investigate how social and environmental factors at different ecological levels (individual, interpersonal, institutional, community, and policy) influence the provision or lack thereof of basic health care in underserved communities in the Philippines. Our findings can be used to develop comprehensive and effective interventions to address the various barriers to health care access and provision and to inform “task shifting” programs and policies in the Philippines and other low- and middle-income countries that aim to encourage and empower communities to take a more active role in health management with the help of BHWs.

### Conclusions

Health professionals, communities, and stakeholders must think outside conventional medicine to improve health [8]. This comprehensive approach must encourage multisectoral cooperation to improve public policy and long-term health care delivery [10]. National, state, and local institutions must coordinate health policy making [4]. In the decentralized Philippines, BHWs could be “local” health experts in multistakeholder dialogues on planning, financing, implementing, managing, and monitoring community health care [9]. Underserved areas need better health education so community members can adopt healthier lifestyles. BHWs need superior training and supervision to provide basic services, health education, and patient referrals. The Philippine Department of Health must provide greater resources to reduce local health promotion barriers [6]. These elements are crucial to health promotion, which creates personal accountability to improve people’s health. Individual and community efforts must modify people’s ideas and habits to enhance health outcomes [9].

## Abbreviations

BHC: barangay health center
BHW: barangay health worker
CHW: community health worker
COREQ: Consolidated Criteria for Reporting Qualitative Research
